# Associations of rs55829688 and rs145204276 Promoter Variants with lncRNA *GAS5* Expression in AML: Prognostic Significance and Functional Analysis

**DOI:** 10.3390/biomedicines14030504

**Published:** 2026-02-25

**Authors:** Djordje Pavlovic, Natasa Tosic, Isidora Curic, Bojan Ristivojevic, Zlatko Pravdic, Nada Suvajdzic Vukovic, Sonja Pavlovic, Branka Zukic, Vladimir Gasic

**Affiliations:** 1 Group for Molecular Biomedicine, Institute of Molecular Genetics and Genetic Engineering, University of Belgrade, 11042 Belgrade, Serbia; djordje.pavlovic@imgge.bg.ac.rs (D.P.); natasa.tosic@imgge.bg.ac.rs (N.T.); isidora.curic@imgge.bg.ac.rs (I.C.); bojan.ristivojevic@imgge.bg.ac.rs (B.R.); sonja.pavlovic99@gmail.com (S.P.); branka.zukic@imgge.bg.ac.rs (B.Z.); 2 Clinic of Hematology, Clinical Center of Serbia, 11000 Belgrade, Serbia; zlatko.pravdic@gmail.com (Z.P.); suvajdzic.nada@gmail.com (N.S.V.); 3 School of Medicine, University of Belgrade, 11000 Belgrade, Serbia

**Keywords:** long non-coding RNA, *GAS5*, acute myeloid leukemia (AML), promoter variants, functional in vitro study

## Abstract

**Background/Objectives**: Acute myeloid leukemia is a genetically diverse hematological malignancy where patient outcomes vary significantly. Long non-coding RNA (lncRNA) *GAS5* acts as a tumor suppressor and is frequently downregulated in various cancers, as well as in AML. In the current study, we aimed to explore the effects of GAS5 promoter variants on its expression levels in AML patients, their prognostic significance, and to investigate their functional effects. **Methods**: The *GAS5* promoter region containing rs55829688 and rs145204276 was sequenced in 75 AML patients. Statistical analyses were performed to assess their associations with *GAS5* expression and outcomes. An in vitro functional study in K562 cells evaluated the effects of these variants on the transcriptional activity of constructs containing each variant. In silico analysis was used to predict changes to transcription factor binding sites. **Results**: Patients carrying the rs55829688 TC/CC genotype exhibited lower *GAS5* expression and were more frequently categorized into the adverse risk group. In intermediate-risk patients, this genotype trended toward lower overall survival and higher bone marrow blast percentages. In vitro, the construct harboring the rs55829688 C allele showed a two-fold decrease in reporter gene activity compared to the construct bearing both wild type alleles. In silico analysis identified RUNX3 as the most likely transcription factor affected by this variant. The variant rs145204276 was considered for the first time in AML; however, no significant clinical associations or transcriptional effects were found. **Conclusions**: Taken together, our findings provide evidence that the rs55829688 promoter variant reduces *GAS5* expression in AML and could potentially be a prognostic marker.

## 1. Introduction

Acute myeloid leukemia (AML) is the most frequently diagnosed form of leukemia in adults, with an incidence rate of over 23,000 cases per year in Europe, and a 5-year survival rate that ranges from 63% in younger patients to only 5% in patients over 70 [1,2]. AML arises from the acquisition of chromosomal rearrangements and mutations affecting genes that regulate normal hematopoietic growth and maturation, leading to an accumulation of immature myeloid precursors in bone marrow. While it can arise from a pre-existing hematological disorder (secondary AML), or as a consequence of treatment of other malignant diseases (therapy-related AML), in most cases, it arises as a de novo malignancy. AML exhibits substantial clinical and genetic diversity, based on which patients can be stratified into certain risk groups and treated accordingly. One of the most widely used stratification methods for patient classification is based on the European LeukemiaNet (ELN) recommendations, classifying patients into favorable-, intermediate-, and poor-risk groups [3]. The discovery of new AML-associated mutations has been steadily refining this classification, but prognosis and outcome still vary significantly, even in patients within the same risk group, in part because the main therapeutic approach remains the same, relying on cytarabine- and anthracycline-based chemotherapy [4].

Nearly half of AML patients do not carry any cytogenetic aberrations, referred to as cytogenetically normal AML (CN-AML). However, in this intermediate-risk group of AML patients, a number of prognostically relevant genetic variants have been identified, such as *FLT3*, *NPM1*, *KIT*, and *IDH1/IDH2* [5]. Additionally, early mutations in genes involved in epigenetic regulation, such as *DNMT3A* and *TET2*, have been shown to lead to pre-malignant clonal hematopoiesis, the risk of which increases with age [6]. Aberrations in these genes, as well as in other genes associated with AML, represent legitimate targets for the design of new therapies, driving towards the expansion of treatment options for AML patients. Since 2017, there have been 11 targeted therapies approved by the FDA, targeting common mutations in genes, such as *IDH1* and *IDH2*, as well as *FLT3* [7]. Even so, many genetic alterations have not yet been incorporated into risk stratification schemes, and have yet to show their potential as markers for better prognosis assessment and personalized therapy approaches [8].

In addition to genetic and epigenetic changes in protein-coding genes, mutations and aberrant expression of non-coding RNAs are further emerging factors involved in cancer development. In particular, long non-coding RNAs, defined as untranslated transcripts longer than 200 nucleotides, play crucial roles in maintaining cellular homeostasis by controlling the cell cycle and regulation of gene expression [9]. *GAS5* (growth arrest-specific transcript 5), named for its increased expression during cell growth arrest, is a lncRNA known to be dysregulated in many types of cancer. *GAS5* achieves its role in tumorigenesis via several mechanisms, such as acting as a decoy for the glucocorticoid receptor, serving as a molecular “sponge” for microRNAs, and regulating translation of proteins [10,11]. Although there is ample evidence that *GAS5* expression is downregulated in solid cancers, the number of studies among hematological malignancies is disproportionately lower, often with contradictory findings. In general, *GAS5* acts as a tumor suppressor across different types of cancer, with its expression downregulated and usually associated with poor prognosis and even resistance to therapy [12]. When it comes to hematological malignancies, in some cases, like AML and diffuse large B-cell lymphoma, *GAS5* is downregulated and associated with poor prognosis, while, in myelofibrosis and childhood acute lymphoblast leukemia, *GAS5* overexpression is a predictor of adverse prognosis and poor therapy [13,14,15,16].

Aberrant expression of *GAS5* is primarily regulated by post-transcriptional mechanisms, namely the mTOR-NMD pathway [17]. However, *GAS5* expression may also be under the control of epigenetic mechanisms, like promoter methylation that can be greatly influenced by the presence of polymorphisms in the promoter region itself, disrupting the methylation process. Particular variants in the *GAS5* gene promoter that affect its expression have also been linked to multiple cancers. For example, the variant rs55829688 is a T > C substitution, 178 base pairs upstream of the *GAS5* transcription start site, which has been associated with decreased *GAS5* expression in colorectal cancer [18]. This variant was also studied in Chinese AML patients and shown to be associated with increased expression of *GAS5* [19]. Another variant, rs145204276, a CAAGG deletion 264 base pairs upstream from the transcription start site, was associated with the decreased expression of *GAS5* in urothelial cell carcinoma, while the same variant was found to increase *GAS5* expression in hepatocellular carcinoma, oral cancer, and renal cell carcinoma [20,21,22,23].

In our previous study, it was shown that *GAS5* expression was downregulated in a group of younger (<65 years) de novo AML patients [13]. In the current study, we aimed to extend our research by exploring the effects of *GAS5* promoter variants on *GAS5* expression levels in AML patients, as well as to investigate their influences in an in vitro functional study in a myeloid setting.

## 2. Materials and Methods

### 2.1. Cohort Clinical Information and Therapy Protocol

We obtained peripheral blood samples from 75 AML patients at disease presentation that were diagnosed and treated at the Clinic of Hematology, Clinical Center of Serbia. Our cohort of patients included 68 cases of de novo AML and 7 cases of secondary AML, diagnosed based on blood blast count over 20% and classified according to the FAB criteria, with the M3 AML subtype excluded. All patients had cytomorphology and immunophenotyping conducted, as well as cytogenetic analysis and molecular analysis of *FLT3* and *NPM1* mutational status. Based on the ELN recommendations, patients were then classified into appropriate prognostic groups. All patients underwent standard 3 + 7 induction chemotherapy with daunorubicin and cytarabine, after which they received three consolidation cycles consisting of high- or intermediate-dose cytarabine. None of the patients received targeted therapies, including FLT3 inhibitors and anti-CD33 drugs. This study was conducted according to the guidelines of the Declaration of Helsinki and approved by the Ethics Committee of the Clinical Center of Serbia, Belgrade, Serbia (No 110/11; Date: 23 May 2019).

### 2.2. GAS5 Promoter Region Genotyping

Genomic DNA was isolated from peripheral blood using the QIAamp DNA Blood Mini Kit (Qiagen, Venlo, The Netherlands), following the manufacturer’s protocol. For the detection of *GAS5* promoter variants, we used polymerase chain reaction (PCR) followed by direct sequencing. Primers were designed for the region upstream of the *GAS5* promoter containing both studied variants—rs55829688 and rs145204276, 86 bps apart (forward: 5′-CCAAAACCCGCAACATTCGC-3′, reverse: 5′-AACACCGTCCCGGAAGTGA-A-3′). The amplification reaction was performed in a total volume of 30 μL, and the reaction mix contained 20 pmol of each primer, 200 ng of genomic DNA, 200 μmol/L of each dNTP (Fermentas, Ontario Canada), 1× PCR reaction buffer (Qiagen), 1× Q solution (Qiagen), 2.75 mM MgCl_2_, and 1 U HotStart Taq DNA polymerase (Qiagen). The temperature profile for the initial activation of DNA polymerase was set at 95 °C for 5 min, followed by 30 cycles of 30 s denaturation at 94 °C, 30 s annealing at 58 °C, and 1 min elongation at 72 °C, ended by a final extension period of 10 min at 72 °C. PCR products of 448 bp were analyzed on 2% agarose gel electrophoresis stained with ethidium bromide and visualized under the UV light. Genotyping was conducted by Sanger sequencing of the PCR product, prepared using the BigDye Terminator v3.1 Cycle Sequencing Kit (Applied Biosystems, Foster City, CA, USA), and performed on the 3130 Genetic Analyzer (Applied Biosystems, Waltham, MA, USA).

### 2.3. Functional In Vitro Study

Three different *GAS5* promoter fragments, generated by PCR and confirmed by Sanger sequencing, were used for functional in vitro assays: the first contained a fragment with the combination of the rs55829688 C allele and rs145204276 wild type, the second contained the combination of rs55829688 wild type and the rs145204276 CAAGG deletion allele, and the third harbored both loci in the wild type state. Each of the fragments was cloned into the pBLCAT5 between the *Hind*III and *Xba*I restriction sites to yield pBLCAT5-GAS5-rs55829688, pBLCAT5-GAS5-rs145204276, and pBLCAT5-GAS5-WT constructs [24]. *E. coli* DH5α strain was used for transformation with the constructs and their amplification. Constructs were isolated using the EndoFree Plasmid Purification Mini Kit (Qiagen, Venlo, The Netherlands). All of the constructs were verified by Sanger sequencing before transient transfections.

K562 cells were maintained in RPMI 1640 medium (Thermo Fisher Scientific, Waltham, MA, USA) supplemented with 10% fetal bovine serum and 100 μg/mL penicillin, streptomycin, and neomycin, at 37 °C in 5% CO_2_ atmosphere. For each transient transfection, 1.6 × 10^6^ K562 cells were plated into 6-well plates and co-transfected with 2 μg of the pBLCAT5-GAS5 constructs, as well as a 2 μg of pCH110 vector (Amersham Pharmacia Biotech, Piscataway, NJ, USA) using Lipofectamine 3000 (Invitrogen, Carlsbad, CA, USA) according to the manufacturer’s protocol. Transfected cells were harvested after 24 h. β-Gal assays were performed with a β-galactosidase enzyme assay system (Promega, Madison, WI, USA), and CAT activities were determined using CAT ELISA (Roche, Basel, Switzerland). CAT activities were normalized using β-Gal activity as an internal control for transfection efficiency, and were evaluated as percentages of wild type activity.

### 2.4. In Silico Analysis of GAS5 rs55829688 and rs145204276 Promoter Variants

An algorithm for predicting the effects of variants investigated in our study on transcription factor binding sites (TFBS), called FABIAN-variant (genecascade.org/fabian/, accessed on 1 January 2026), was used to predict which transcription factors (TFs) bind to particular rs55829688 and rs145204276 sites on the *GAS5* promoter region and their potential functional effects on *GAS5* expression [25]. The obtained potential TFs were filtered to include only known TFBSs using the JASPAR database as a source [26]. Prediction scores of TFs whose binding sites were affected by gain or loss of function were then cross-checked with the Genotype-Tissue Expression (GTEx, gtexportal.org) Analysis Release V10 to obtain their predicted expression levels in blood, in transcripts per million. The prediction scores and expression levels were combined to assess the most likely transcription factor to affect *GAS5* expression.

### 2.5. Statistical Analysis

Data on clinical characteristics of AML patients (Table 1) are presented as medians with range, or as absolute numbers, with percentages in brackets. Overall survival (OS) was calculated from the first day of therapy to death or last visit. Patients undergoing hematopoietic stem cell transplantation (HSCT) were censored at the time of transplantation.

Differences in continuous variables were analyzed using the Student’s *t*-test for distribution between two groups. Frequency analyses were performed using the χ^2^ test for 2 × 2 tables, or the Fisher exact test for larger tables. Survival analysis was performed by the Kaplan–Meier method, and differences in survival distributions were evaluated using the Log-Rank test. The statistical software used was R software, version 4.4.0.

## 3. Results

### 3.1. Associations of GAS5 Promoter Variants and Expression Levels in AML Patients

**Figure 1:**
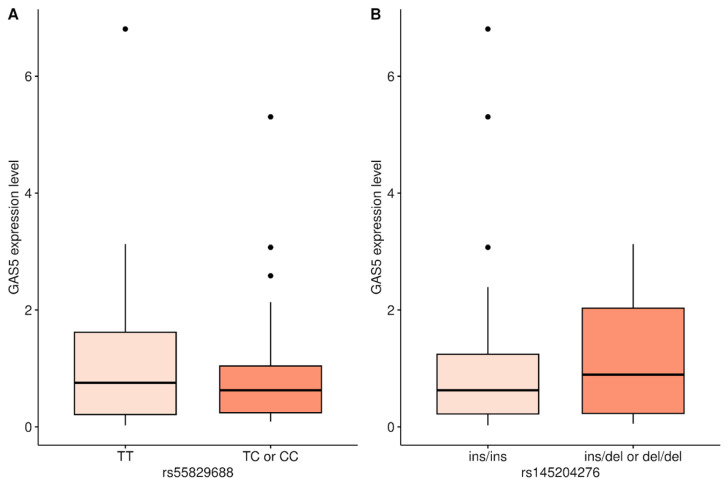
(**A**) *GAS5* expression levels in AML patients carrying the rs55829688 TC/CC and TT genotypes (*n* = 42 vs. 33, median 0.566 vs. 0.706, *p* = 0.028 without outliers, *p* = 0.531 with outliers). (**B**) *GAS5* expression levels in patients carrying the rs145204276 ins/ins and ins/del or del/del genotypes (*n* = 53 vs. 22, median 0.892 vs. 0.625, *p* = 0.08 without outliers, *p* = 0.538 with outliers).

*GAS5* expression results reported in our previous study were used to compare the effects of two promoter variants on *GAS5* expression levels in 75 AML patients [13]. For the rs55829688 variant, patients were grouped according to a dominant model into those carrying only the T allele (TT genotype), and those carrying at least one C allele (TC/CC genotype), as there was no significant difference between the expression levels of patients with the TC and CC genotypes, as well as the small number of CC genotype patients. After adjusting for outliers, patients with the TC/CC genotype showed a significantly lower *GAS5* expression level compared to those with the TT genotype group (median 0.566 vs. 0.706, Figure 1A, *p* = 0.028 without outliers, *p* = 0.531 with outliers). For the rs145204276 variant, patients were similarly grouped according to a dominant model into those carrying only the wild type allele (ins/ins genotype), and those carrying at least one CAAGG deletion allele (ins/del or del/del genotype), but no significant difference in *GAS5* expression level was found (median 0.892 vs. 0.625, Figure 1B, *p* = 0.08 without outliers, *p* = 0.538 with outliers).

### 3.2. Association of GAS5 Promoter Variants with the Clinical Features and Prognosis of AML

The association of the clinical features of AML patients with *GAS5* variant genotypes is summarized in Table 1. The rs55829688 TC/CC genotype was more likely to be found in patients with an adverse risk prognosis (70% TC/CC genotype, *p* = 0.018). Though the platelet count in patients with a rs145204276 ins/ins genotype was lower (median 42 × 10^9^/L vs. 79 × 10^9^/L for ins/del and del/del), and the bone marrow blast percentage was higher in patients with a rs55829688 TC/CC genotype (median 59.5% vs. 70% for TT), these results did not reach statistical significance (*p* = 0.074 and *p* = 0.083, respectively).

### 3.3. The Roles of GAS5 Promoter Variants in the Prognosis of Intermediate-Risk Patients

**Figure 2:**
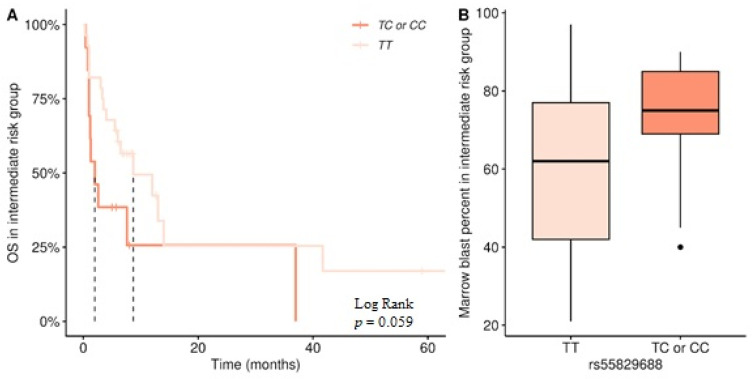
(**A**) Kaplan–Meier analysis of overall survival (OS) in intermediate risk AML patients according to rs55829688 genotype (TT: *n* = 28, 10 censored, TC/CC: *n* = 13, 3 censored, median 8.7 vs. 2 months, Log-Rank = 3.6, *p* = 0.059) (**B**) Difference in marrow blast levels in intermediate-risk patients with rs55829688 TT and TC/CC genotypes (median 62 vs. 75 percent in the TT genotype, *p* = 0.052). The dashed lines designate the median values of survival.

Due to the fact that the intermediate group is the most numerous, and the most heterogeneous in terms of outcome, we wanted to test whether the *GAS5* promoter variants could be used to better stratify the intermediate-risk group. In this group, patients with the rs55829688 TC/CC genotype had a lower OS compared to the TT genotype (2 vs. 8.7 months median, Log-Rank = 3.6, Figure 2A), bordering on statistical significance (*p* = 0.059). Also, an increased percentage of bone marrow blasts as a marker of poor prognosis showed a similar trend, being higher in patients with the TC/CC genotype (median 75% vs. 62% in the TT genotype, Figure 2B, *p* = 0.052).

### 3.4. Functional Analysis of GAS5 Promoter Variant Constructs in K562 Cells

**Figure 3:**
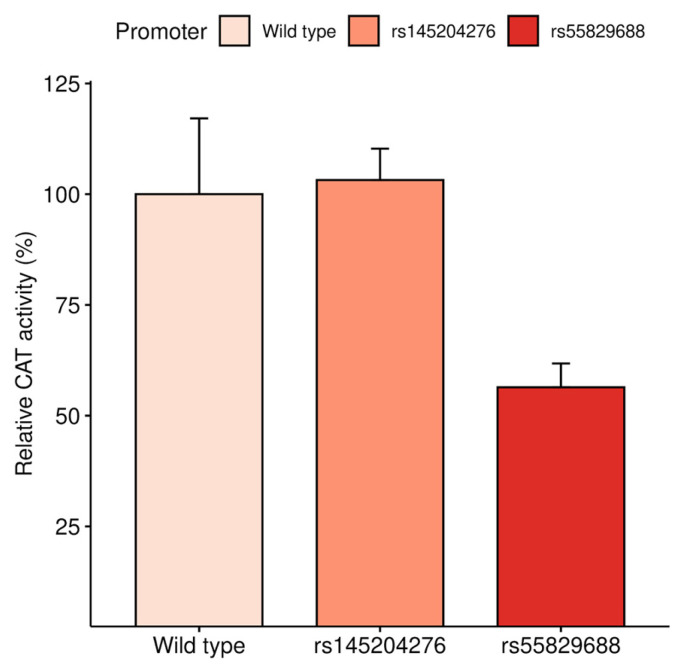
The transcriptional activity of the *GAS5* promoter fragments carrying different variants in K562 cells using CAT reporter gene assay (*p* = 0.041).

To determine the effects of the two studied *GAS5* promoter variants on transcriptional activity in vitro in a myeloid leukemia model system, reporter gene assays were performed on the permanent K562 cell line transiently transfected with three plasmid constructs: carrying the rs55829688 T > C variant alone, rs145204276 CAAGG deletion variant alone, and a control construct carrying both wild type alleles. Results of the reporter gene assay showed that the reporter gene activity of the construct carrying the rs55829688 C allele was decreased nearly two-fold compared to the activity of the control plasmid carrying the wild type alleles and the one carrying only the rs145204276 CAAGG deletion allele (Figure 3, *p* = 0.041).

An algorithm that predicts variant effects on known TFBSs, called FABIAN-variant, was used to predict the effect of the rs55829688 T > C variant and assess the possible cause of the decrease in transcriptional activity detected by the in vitro assay. Prediction scores obtained by FABIAN-variant, combined with the average expression levels of the predicted TFs in blood from the GTEx public database, revealed that the most likely candidate TFBS affected by the rs55829688 C allele is RUNX3. RUNX3 is both highly expressed in blood and has the third highest score for TFBS loss when rs55829688 is present (Table 2).

## 4. Discussion

The downregulation of *GAS5* is a hallmark of many types of solid tumors, affecting clinical features such as tumor size and stage, as well as lymph node invasion and metastasis [10]. In our previous study, it was shown that *GAS5* expression is lowered in AML patients and is associated with an adverse disease risk [13]. *GAS5* level can also affect therapy response, as it does in acute lymphoblastic leukemia through its effects on glucocorticoids [27,28]. A possible explanations for the lower *GAS5* expression level could be the presence of variants in the region upstream of the promoter, including the rs55829688 variant analyzed in our study [29].

The variant rs55829688 T > C in the promoter region of *GAS5* was examined in AML patients using data from a GEO public dataset, where the authors showed that the CC genotype is associated with elevated *GAS5* expression, likely due to the increased binding affinity of the TP63 transcription factor [19]. In contrast, our results showed that AML patients carrying the rs55829688 C allele exhibit significantly lower *GAS5* expression after adjusting for outliers. This is in line with findings reported for colorectal cancer, which showed that patients carrying the rs55829688 CC genotype displayed lower *GAS5* expression due to the C allele exhibiting lower affinity for YY1 [12,18]. This variant has also been investigated in non-cancer studies. The rs55829688 C allele was reported to be associated with lower *GAS5* expression in advanced diabetic kidney disease patients [30]. The *GAS5* rs55829688 variant may also alter clopidogrel efficacy in coronary artery disease patients with CYP2C19 poor-metabolizer genotypes by regulating P2Y12 expression through sponging miR-223-3p [31]. Carriers of the rs55829688 C allele may be associated with a lower risk of systemic lupus erythematosus [32]. These findings demonstrate that *GAS5* is a critical regulator of multiple signaling pathways and gene expression in diverse pathophysiological settings.

In our cohort, adverse risk AML patients were more likely to be rs55829688 C allele carriers, although this was not associated with clinical features in the overall group. Given the significant prognostic importance of proper classification of patients into risk groups, we investigated whether rs55829688 T > C could be used to further stratify the AML intermediate-risk group. In the present study, a trend that rs55829688 C allele carriers had a lower overall survival rate was detected. This was further supported by the result that those patients exhibited higher bone marrow blast percentage, a feature well-established as a worse prognostic factor in AML [33]. These results add further value to the understanding of the tumor suppressor role of *GAS5* in AML and are consistent with studies that have shown that rs55829688 T > C is a possible contributor to lower expression [34].

The evidence that the rs55829688 C allele might affect the regulation of *GAS5* expression in AML is further supported by the results of our functional analysis, which showed decreased reporter gene activity of the construct carrying the rs55829688 C variant in comparison to the construct bearing both wild type alleles in K562 myeloid cells. This finding is in line with results shown in human colorectal cancer cell lines [18]. To suggest the possible cause of this observation, we obtained a list of potential transcription factors, whose binding sites would be affected by the variant, and compared their prediction scores with the average expression levels of these transcription factors in blood, ending up with RUNX3 as the most likely candidate responsible for the transcriptional decrease [25]. In the abovementioned study on colorectal carcinoma, it was suggested that the reduced expression of *GAS5* occurs because the rs55829688 variant alters the binding affinity of the transcription factor YY1 to *GAS5* [18]. We have also identified YY1 as a potential TF that could be responsible for the decrease in *GAS5* expression; however, our in silico analysis revealed that RUNX3 is more highly expressed in blood, suggesting possible tissue specificity. The RUNX family of transcription factors are known participants in hematopoietic development, with RUNX3 in particular playing a crucial role in lineage-specific differentiation of lymphocytes, leading to myeloproliferative disorders in deficient mice [35,36,37]. RUNX3 is also often a target of suppression in multiple cancers, including hematological malignancies [38]. Interestingly, RUNX3 was recently connected to *GAS5* signaling in liver cancer, where it was shown that *GAS5* is downregulated in the NK cells of liver cancer patients, leading to a downregulation of RUNX3 through the upregulation of miR-544, which was associated with a decrease in NK cell function. It is possible that this axis is further impaired by the loss of the RUNX3 binding site. Overexpression of *GAS5*, on the other hand, led to enhancement of NK cell activity and decreased tumor volume through RUNX3 upregulation, indicating that *GAS5* could be a potential target for NK cell-based immunotherapy [39].

Another *GAS5* promoter variant, which is well studied in solid tumors, is the rs145204276 CAAGG deletion variant, but it has seemingly not been studied before in hematological malignancies. This variant has been associated with an increase in *GAS5* expression, which led to a reduced risk of developing renal cell carcinoma [23]. A similar protective role was shown in gastric cancer, possibly owing to the interactions of *GAS5* with E2F1 and CDKN1B, while, in prostate cancer, it contributed to higher *GAS5* expression and better clinical outcome when combined with rs55829688 [40,41]. In osteosarcoma, on the other hand, it was shown that rs145204276 is associated with disease susceptibility, with the del/del genotype showing higher *GAS5* expression, likely by influencing promoter methylation [42]. Similarly, this variant also led to increased *GAS5* expression in hepatocellular carcinoma by affecting the methylation status of the promoter, and it was correlated with an increased risk of developing the disease, although this is at odds with other *GAS5* studies in hepatocellular carcinoma [21,43,44]. This variant was also associated with higher *GAS5* expression and worse tumor stage in oral cancer, leaving an open question as to a possible proto-oncogenic role of *GAS5* in certain cancers [22]. Our study revealed no significant difference in *GAS5* expression level in AML patients who carry the rs145204276 deletion allele, and, to the best of our knowledge, there are no other studies connecting this variant with leukemia. The results of our functional analysis that the transcriptional activity of the rs145204276 deletion allele does not differ from the wild type allele is not in line with previous functional in vitro studies. In a study investigating the association of this allele with an increased risk of ischemic stroke, it was shown that rs145204276 leads to an elevated *GAS5* expression level in HEK293 cells, suggesting that its effect may be tissue specific [45]. All this goes in favor of supporting the idea that further study is needed, with the aim of understanding the potential role of this variant in hematological malignancies.

## 5. Conclusions

Long non-coding RNAs have been discovered as potential regulators of many signaling pathways and gene expression levels in diverse pathophysiological settings. *GAS5* expression as a prognostic factor was investigated in many clinical entities, and its potential is not fully explored. Our results showed that *GAS5* promoter variant rs55829688 is associated with lower *GAS5* expression in AML patients, but its unfavorable impact on clinical outcome was visible only in the group of patients with intermediate risk. Furthermore, a functional in vitro study in myeloid K562 cells supported this finding, revealing a two-fold decrease in the expression of *GAS5* promoter construct bearing the rs55829688 variant. In silico analysis revealed that the RUNX3 transcription factor could be a tissue-specific factor binding to the rs55829688 variant site. We believe that the results of this study contribute to the comprehensive knowledge of *GAS5* in AML, but studies based on larger numbers of patients and using wider perspectives on the importance of *GAS5* as a marker of pathogenesis and prognosis are necessary.

## Figures and Tables

**Table 1:** Clinical characteristics of AML patients stratified by *GAS5* variant genotype.

Parameter		rs55829688			rs145204276		
	Total *n* = 75	TT *n* = 42	TC or CC *n* = 33	*p*	ins/ins *n* = 53	ins/del or del/del, *n* = 22	*p*
**Sex**				0.646			0.265
Male (%)	42	25 (60)	17 (40)		27 (36)	15 (20)	
Female (%)	33	17 (52)	16 (48)		26 (35)	7 (9)	
**Age**, years median (range)		50 (18–62)	49 (18–61)	0.399	50 (18–62)	49.5 (18–61)	0.798
**WBC count**, ×10^9^/L median (range)		16.25 (1–258.8)	19 (1.4–348.8)	0.509	20.9 (1.1–348.8)	4 (1–183.7)	0.22
**Hemoglobin** (g/L) median (range)		97.5 (2–166)	98 (65–153)	0.517	97 (71–153)	102.5 (2–166)	0.514
**Platelets** (×10^9^/L) median (range)		53.5 (2–216)	49 (1–169)	0.736	42 (1–216)	79 (8–180)	0.074
**LDH** (U/L) median (range)		212 (175–4169)	357 (175–2147)	0.718	357 (175–4169)	175 (175–1314)	0.097
**Peripheral blood blast** (%) median (range)		16 (0–98)	22 (0–97)	0.413	20 (0–97)	15 (0–98)	0.167
**Bone marrow** blasts (%) median (range)		59.5 (20–97)	70 (24–94)	0.083	69 (20–97)	58 (21–87)	0.229
**FAB** (%)				0.286			0.09
M0	6	4 (67)	2 (33)		3 (50)	3 (50)	
M1	9	5 (56)	4 (44)		4 (44)	5 (56)	
M2	19	8 (42)	11 (58)		12 (63)	7 (37)	
M4	27	19 (70)	8 (30)		23 (85)	4 (15)	
M5	14	6 (43)	8 (57)		11 (78)	3 (22)	
**Karyotype** (%)				0.533			1.0
Normal Karyotype (NK)	36	22 (61)	14 (39)		25 (69)	11 (31)	
Other	39	20 (51)	19 (49)		28 (61)	11 (39)	
**NPM1 in NK** (%)				0.303			0.722
Present	13	6 (46)	7 (54)		10 (77)	3 (23)	
Absent	23	16 (69)	7 (31)		15 (55)	8 (45)	
**FLT3 in NK** (%)				0.430			0.834
Present	9	4 (44)	5 (56)		7 (78)	2 (22)	
Absent	27	18 (75)	9 (25)		18 (75)	9 (25)	
**ELN risk group** (%)				**0.018**			0.521
Favorable	14	8 (57)	6 (43)		10 (72)	4 (28)	
Intermediate	41	28 (68)	13 (32)		27 (65)	14 (35)	
Adverse	20	6 (30)	14 (70)		16 (70)	4 (30)	
**Complete remission** (%)				0.608			0.906
Success	40	16 (40)	24 (60)		29 (72)	11 (28)	
Failure	35	17 (48)	18 (52)		24 (69)	11 (31)	
**Relapse** (%)				0.846			0.302
Yes	22	14 (64)	8 (36)		14 (64)	8 (36)	
No	18	10 (55)	8 (45)		15 (83)	3 (17)	

**Table 2:** The predicted effect of rs55829688 on known TFBSs according to FABIAN-variant, and the expression levels in transcripts per million of those transcription factors in blood.

Transcription Factor	Effect on TFBS (Fabian)	Expression Level in Blood (TPM)
SP4	**−0.4769**	Low (1.304)
YY1	**−0.3370**	Low (14.68)
**RUNX3**	**−0.3138**	**High (33.07)**
NR2F1	−0.1234	Low (<1)
EGR1	−0.1127	Low (2.938)
NFIC	−0.0727	Low (5.630)
STAT3	0.0569	**High (101.6)**
E2F4	0.0876	**High (62.69)**
ARNT	0.1155	Low (17.18)
GMEB1	0.1798	Low (3.975)

## Data Availability

The authors confirm that the data supporting the findings of this study are available within the article; further inquiries can be directed to the corresponding author.

## Abbreviations

The following abbreviations are used in this manuscript:

AML	Acute myeloid leukemia
ELN	European LeukemiaNet
lncRNA	long non-coding RNA
TFBS	Transcription factor binding site
